# Awake Tracheal Intubation Using the ProVu™ Video Stylet in a Patient With Difficult Airway Due to Acromegaly

**DOI:** 10.7759/cureus.106905

**Published:** 2026-04-12

**Authors:** Sebastián Monsalves, Juan Pablo Borgoño, Natalia Roman

**Affiliations:** 1 Department of Anesthesiology, Clínica Alemana - Universidad del Desarrollo, Santiago, CHL; 2 Faculty of Medicine, Clínica Alemana - Universidad del Desarrollo, Santiago, CHL

**Keywords:** awake video stylet intubation, device for awake intubation, difficult airway management, intubation in a patient with acromegaly, provu video stylet in difficult airway management

## Abstract

Acromegaly is characterized by progressive craniofacial and soft-tissue changes that can complicate airway management. Awake tracheal intubation (ATI) is recommended when a difficult airway (DA) is anticipated, particularly when the loss of spontaneous ventilation poses substantial risk. We report the case of a patient with acromegaly secondary to a pituitary macroadenoma who underwent transsphenoidal surgery. Preoperative awake airway assessment revealed severe upper airway narrowing, reduced nasal patency, and glottic restriction, which warranted the use of awake tracheal intubation. The airway was successfully secured using the ProVu™ Video Stylet (Flexicare Medical Ltd., Mountain Ash, United Kingdom) after topical anesthesia and light conscious sedation. The device facilitated the controlled advancement of the endotracheal tube under continuous visualization despite marked anatomic distortion. This case highlights the potential role of a steerable video stylet as an alternative to flexible bronchoscopy for patients with difficult airway anatomy.

## Introduction

Acromegaly, most commonly caused by growth hormone (GH)-secreting and insulin-like growth factor-1 (IGF-1)-secreting pituitary adenomas, is associated with progressive multisystem morbidity. Upper airway involvement is particularly relevant in perioperative care, as soft-tissue hypertrophy and craniofacial overgrowth may result in macroglossia, prognathism, pharyngeal narrowing, and laryngeal distortion [1]. These anatomic changes increase the likelihood of difficult airway (DA) management and may contribute to obstructive sleep apnea syndrome (OSAS) [2].

Awake tracheal intubation (ATI) is an essential strategy when difficult intubation is anticipated, when apnea is poorly tolerated, or when the likelihood of successful supraglottic rescue is uncertain [3]. Historically, flexible bronchoscopy has been the standard technique; however, videolaryngoscopy and video stylets have demonstrated comparable efficacy and safety in adults [4]. Video stylets offer practical advantages, including the ease of use, faster intubation in experienced hands, and a shorter learning curve. In addition, certain designs, such as the ProVu™ Video Stylet (Flexicare Medical Ltd., Mountain Ash, United Kingdom), enable the operator to guide endotracheal tube advancement under continuous visualization, thereby improving the control of the tube’s passage through the airway.

Video stylets have emerged as a useful alternative, combining direct visualization with enhanced control over the endotracheal tube’s trajectory [5].

The ProVu™ Video Stylet is a rigid, malleable intubation device with an actively steerable flexible tip and a miniature camera at its distal end. This configuration may be advantageous in anatomically distorted airways, enabling real-time navigation while maintaining control over tube direction. We describe its use for ATI in a patient with severe acromegaly-related airway changes undergoing transsphenoidal pituitary surgery.

## Case presentation

A 60-year-old woman, weighing 71 kg and measuring 158 cm in height (BMI: 28 kg/m²), with a history of hypertension and acromegaly secondary to a functioning pituitary macroadenoma, had a long-standing history of snoring and apnea. Table 1 summarizes the findings of the preoperative airway assessment, which suggested a potentially difficult airway.

**Table 1:** Preoperative airway assessment

Parameter	Result
Oral cavity	Opening: 45 mm; macroglossia
Cervical mobility	Reduced cervical flexion and extension
Mallampati score [6]	4
Bite	Prognathism with a class III bite
Neck circumference	40 cm
Thyromental distance	9 cm
Sternomental distance	16 cm
Submandibular space	Compliance reduced
Cricothyroid membrane	Not palpable
Nasal cavity	Narrow and obstructed by turbinate hypertrophy

Preparation for perioperative evaluation and awake intubation was conducted in the operating room with two anesthesiologists, one attending and one resident, following the layout and ergonomic recommendations of the Difficult Airway Society guidelines on ATI [7]. The patient was positioned in a semi-sitting position at 45°. Monitoring included invasive right radial arterial pressure, three-lead electrocardiography, pulse oximetry, capnography, inline oxygen monitoring, temperature, and a Foley catheter.

**Figure 1:**
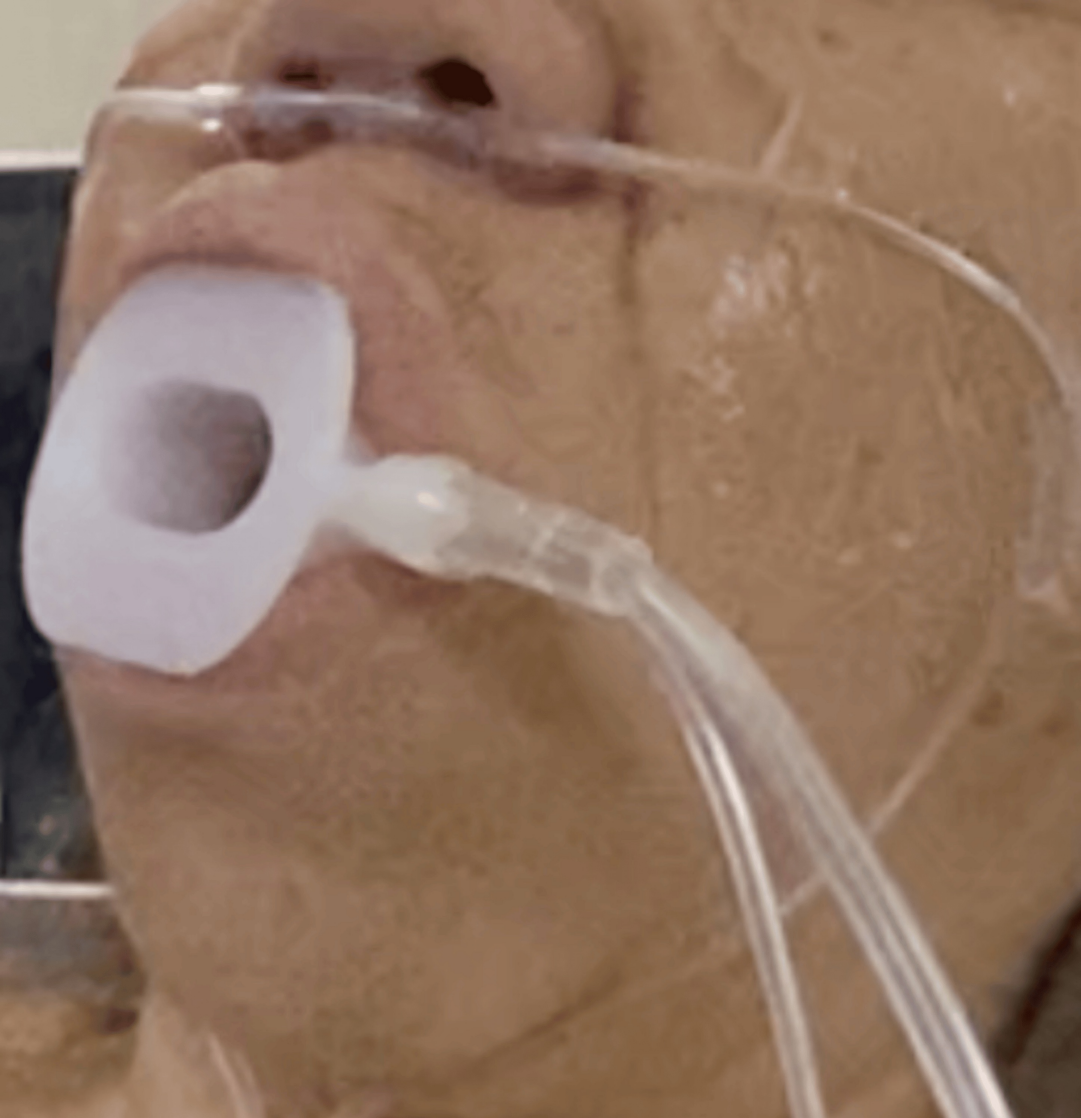
Preoxygenation settings Nasal cannula oxygen at 2-3 L/minute combined with the M1 endoscopic mouthpiece at 15 L/minute

**Figure 2:**
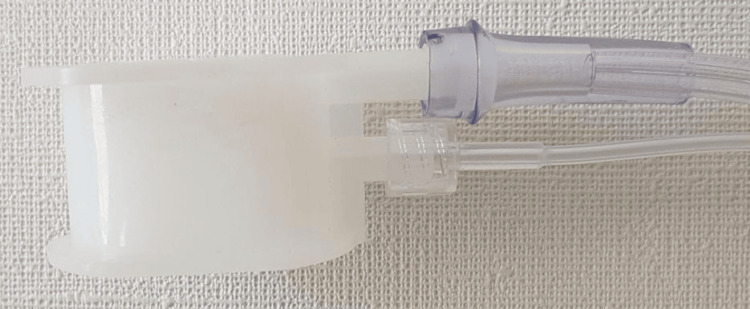
M1 endoscopic mouthpiece (SIOL®) The M1 endoscopic mouthpiece is used for awake airway management. This multifunctional device facilitates oxygen administration and capnography attachment while maintaining oral access for endoscopic procedures and airway instrumentation. In this case, it is employed for oral preoxygenation and video stylet insertion. The image is used with the permission of its creator, Dr. Cristian Muñiz, anesthesiologist at the Hospital del Trabajador-Asociación Chilena de Seguridad (ACS), Santiago, Chile

Dual-route oxygenation was employed (Figure 1). Supplemental oxygen was administered at 3 L·minute^-1^ via nasal cannula, with simultaneous oral insufflation through the endoscopy mouthpiece (M1) (Figure 2) to maintain adequate periprocedural oxygenation.

Airway topicalization included 1 mL of 10% lidocaine applied to the palatopharyngeal and palatoglossal pillars and 4 mL of 2% lidocaine gel applied to the nasal vestibule.

An additional 1% lidocaine was applied to the left nasal fossa, nasopharynx, and oropharynx using the Laryngo-Tracheal Mucosal Atomization Device (LMA MADgic) (Teleflex, Wayne, PA) and to the glottis and trachea via the working channel of the video bronchoscope [8].

The total dose of lidocaine was 380 mg, equivalent to 8.6 mg/kg of lean body mass, which was within the safety range recommended in the Difficult Airway Society guidelines on ATI.

Light sedation was achieved with dexmedetomidine infused at 0.75 mcg/kg/hour and remifentanil, initially titrated at 0.15 mcg/kg/minute and subsequently maintained in effect-site target-controlled infusion mode at 4.0 ng/mL using the Minto model.

Awake diagnostic airway assessment was performed before intubation. An initial videolaryngoscopy with a C-MAC D-Blade (Karl Storz, Tuttlingen, Germany) was performed to assess glottic exposure and oropharyngeal working space. Despite the correct placement of the blade in the glossoepiglottic sulcus, adequate epiglottic elevation could not be achieved, and the arytenoids could not be identified. Furthermore, the insertion of the blade substantially reduced the available oropharyngeal space, suggesting that tube manipulation with a semi-rigid introducer or bougie would be challenging.

A complementary endoscopic examination was performed with the patient awake using a Karl Storz FIVE 4.0 (Tuttlingen, Germany) video bronchoscope and revealed nasal obstruction due to polyposis, chronically narrowed nasal passages with septal deviation, and nasopharyngeal collapse. The epiglottis was thickened, shortened, and in close apposition to the posterior oropharyngeal wall. The lateral oropharyngeal walls were thickened and showed expiratory collapse. The arytenoids and vocal cords were enlarged, and the glottic inlet was narrow. Advancement toward the trachea required a pronounced flexion-deflection maneuver, indicating substantial angulation between the primary and secondary airway curves.

**Figure 3:**
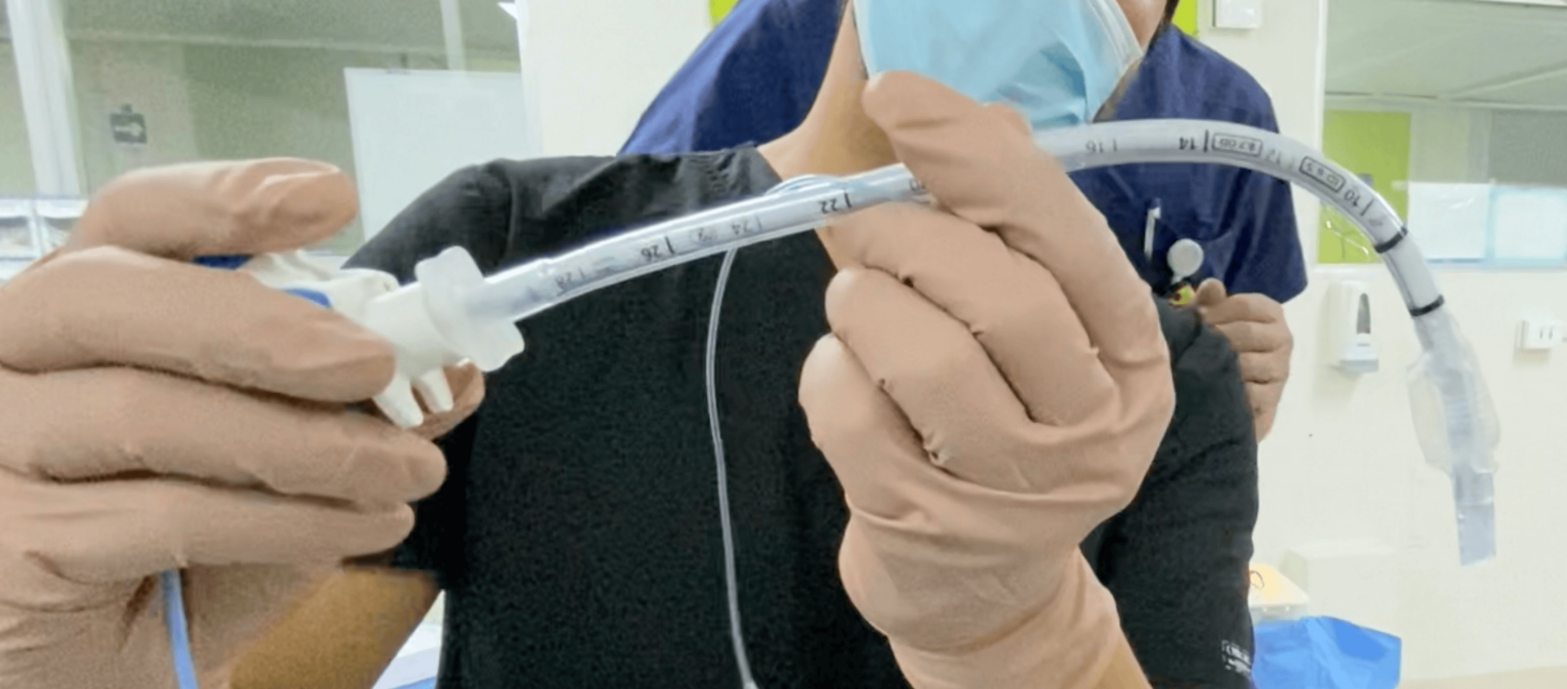
ProVu™ Video Stylet pre-shaped to 90°

Based on these findings, the ProVu™ Video Stylet was pre-shaped to a 90° angle, with further adjustment of the malleable distal tip as necessary (Figure 3). ATI was then performed orally through the M1 endoscopic oral device using a 7.0 mm reinforced endotracheal tube mounted on the stylet. The preconfigured shape allowed the prompt identification of the epiglottis, and sequential flexion-deflection maneuvers positioned the tube beneath the epiglottis and then through the vocal cords under direct vision. Upon entry into the trachea, the semi-rigid malleable guidewire was removed, and the stylet tip was deflected to avoid contact with the inner surface of the cricothyroid membrane, facilitating smooth, atraumatic advancement. Tracheal rings were clearly visualized, and the tube was positioned approximately 2-3 cm above the carina. The cuff was inflated while the patient remained awake, and correct placement was confirmed by capnography before the induction of general anesthesia (Video 1).

**Video 1:** Awake tracheal intubation using the ProVu™ Video Stylet

**Figure 4:**
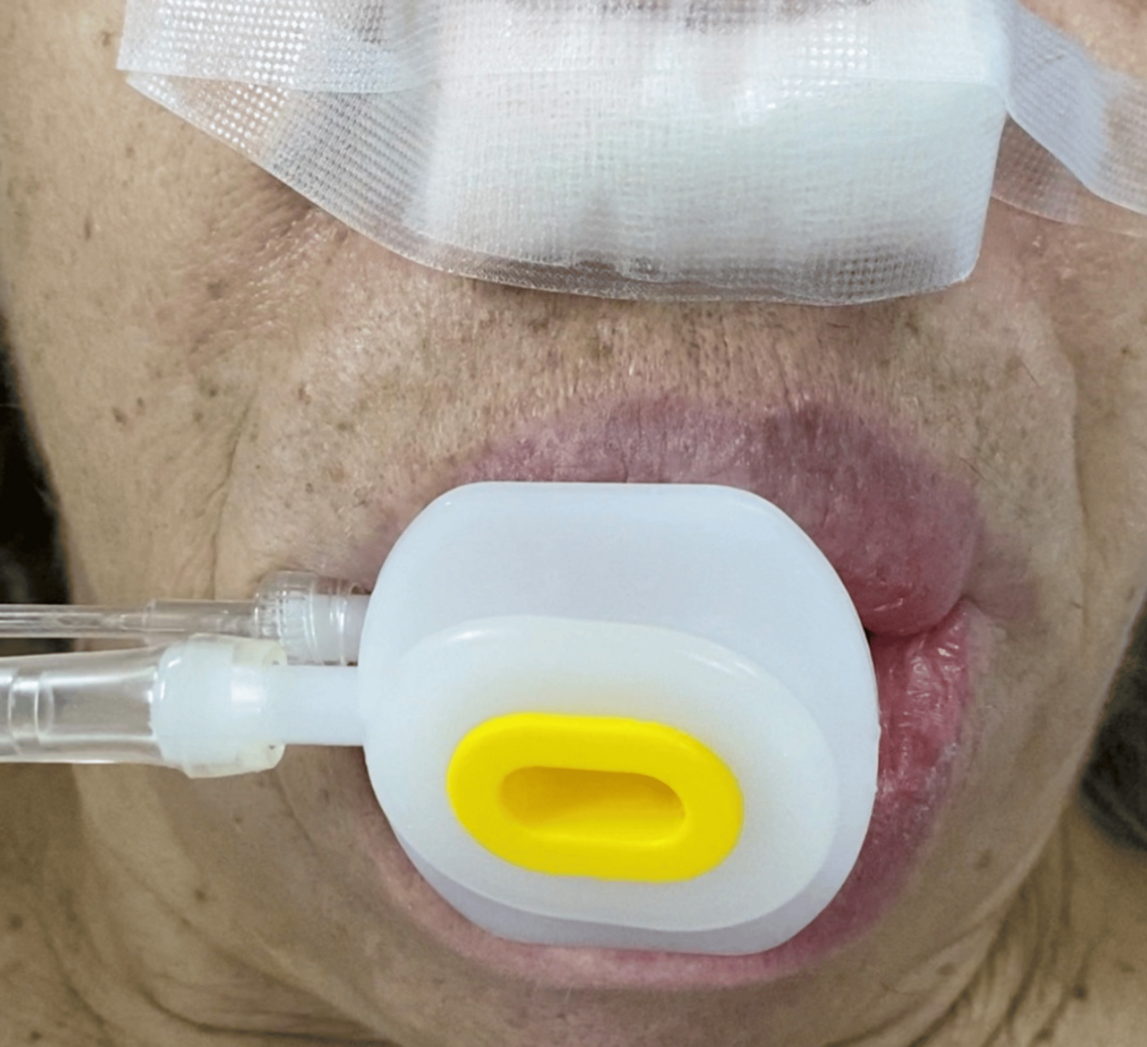
M1 endoscopic mouthpiece (SIOL®) delivering oxygen at 15 L/minute and a size 3 Guedel airway for safe extubation

The patient was extubated in the semi-sitting position with remifentanil support at a target concentration of 2.0 ng/mL and supplemental oxygen delivered through an M1 mouthpiece, together with a size 3 Guedel airway (Figure 4). Extubation was calm and well-tolerated. No desaturation episodes were recorded.

## Discussion

ATI remains the recommended primary strategy in patients with anticipated DA, particularly when there is intolerance to apnea, a risk of aspiration, or uncertainty regarding supraglottic rescue [9]. It is also highly beneficial in patients with head and neck tumors and in cases of restricted mouth opening, where conventional laryngoscopy may be ineffective or risky [10].

ATI can be performed using virtually all devices that facilitate tracheal intubation, including the Macintosh laryngoscope, videolaryngoscopes, video stylets, and flexible bronchoscopes. Less common approaches, such as intubation through supraglottic devices, retrograde techniques, or blind nasal intubation, may also be used [11]. Historically, flexible bronchoscopy has been considered the gold standard for ATI, with success rates nearing 98%-99% and a well-established safety profile [12]. However, its performance decreases in the presence of glottic edema, substantial anatomic distortion, or an inability to advance the tube under direct vision, conditions that were all present in our case [13].

Conversely, videolaryngoscopy has demonstrated comparable efficacy in adults, offering advantages such as shorter intubation time and lower desaturation rates [14]. However, its success depends on adequate mouth opening, proper blade insertion, and submandibular compliance. The ability to create sufficient working space in the oropharynx for tube passage is critical; thus, macroglossia and limited submandibular compliance are substantial limiting scenarios. Operator expertise is also pivotal, as training directly influences success rates [15].

In our patient, videolaryngoscopy with the C-MAC D-Blade did not provide an adequate glottic view. The exposure was partial, which indicated the need for forceful maneuvers, such as further deflection, higher pressure, and deeper insertion, posing a risk of injury to friable mucosa and compromising patient tolerance. Practically, the blade’s handle-to-tip distance was insufficient, hindering normal insertion. According to Greenland’s curve theory, the blade geometry did not match the primary airway curvature, which, combined with the limited oropharyngeal space, introduced a risk of trauma and intubation failure [16].

In this case, flexible bronchoscopy also had limitations. The inability to advance the tube under direct vision posed a substantial risk of trauma in the setting of arytenoid hypertrophy and a narrow glottis. Additionally, the marked angulation of the tube trajectory from the glottis to the trachea necessitated an airway management strategy that would provide the visualization of tube passage while accommodating the airway trajectory.

Videolaryngoscopy-assisted fiberoptic intubation (VAFI) offers a reasonable alternative in this context [17,18]. The videolaryngoscope provides a panoramic view and allows the visualization of tube passage through the glottis, while the flexible bronchoscope facilitates adaptation to the airway trajectory.

Desai et al. demonstrated that optical stylets have efficacy and safety comparable to videolaryngoscopy and fiberoptic bronchoscopy in ATI and were associated with the shortest intubation times, supporting their consideration as a valid alternative in DA [19].

In 2021, Harrison et al. reported three cases in which the ProVu™ Video Stylet was decisive [20]. In all cases, videolaryngoscopy and flexible bronchoscopy were considered inadequate: videolaryngoscopy due to limited working space and restricted mouth opening and bronchoscopy because it could not mobilize collapsed soft tissues or solid tumors. In all three cases, the ProVu™ Video Stylet adapted to the primary curvature and facilitated successful first-attempt awake intubation without complications. Similarly, in our case, the C-MAC D-Blade was not advantageous because of macroglossia and minimal submandibular compliance, while videobronchoscopy suggested a risk of arytenoid trauma. Therefore, the hybrid VAFI approach was not feasible, and ATI with ProVu™ was a reasonable alternative.

During the procedure, the ProVu™ allowed for straightforward awake intubation. Its rigidity was sufficient to mobilize the epiglottis, and its range of motion facilitated alignment with the desired trajectory. Tube passage was controlled, with high-quality imaging and no fogging despite the presence of secretions. Although the learning curve for ATI has not been formally described, it is likely shorter than that for flexible bronchoscopy, given that operators are generally familiar with conventional stylets. Desai et al.’s systematic review and network meta-analysis also reported that video stylets have success and complication rates comparable to those of flexible bronchoscopy and videolaryngoscopy while achieving shorter intubation times.

Regarding the bidirectional wheel-controlled tip, handling was intuitive because its directional control is similar to that of flexible bronchoscopes, facilitating skill transfer. Disadvantages include a smaller flexion/deflection range (35°/60°) than that of flexible optics (180°/130°), the absence of a working channel for suction or oxygen delivery, and the restriction to factory-preloaded tubes, which limits its use to the adult population.

## Conclusions

The ProVu™ Video Stylet may be considered a useful alternative for ATI in selected difficult airway cases, particularly when the simultaneous control of tube passage and trajectory guidance is required. In this case, it allowed successful intubation under direct vision despite anatomic features that limited both videolaryngoscopy and flexible bronchoscopy. Although additional evidence is needed, this experience suggests that the ProVu™ Video Stylet may be a reasonable option within the ATI armamentarium.
